# ‘You Are Not Alone, We’ve Got You’: Power Plays, Devotion, and Punishment on Healthy Eating and Pro-Eating Disorder Websites

**DOI:** 10.1177/10497323241238628

**Published:** 2024-03-26

**Authors:** Panagiota Tragantzopoulou, Alison Fixsen, Damien Ridge, Anna Cheshire

**Affiliations:** 1 School of Social Sciences, 4921 University of Westminster, London, UK

**Keywords:** healthy eating websites, pro-eating disorder websites, healthy eating, anorexia, discourse analysis

## Abstract

Healthy eating (HE) and pro-eating disorder (pro-ED) websites are popular sources of dietary and weight loss information, social support, and lifestyle inspiration. However, the discursive styles and language used by authors/moderators and users of these two site genres have not been widely studied or compared. Forty-three HE websites and twenty-four pro-ED websites were analysed using Fairclough’s model of critical discourse analysis. Findings indicate that sites share common characteristics in terms of power relations played out by authors, ‘successful’ dieters, and those attending these sites. These power plays encourage moral and spiritual commitment to the care of one’s body, with authoritative language used to support readers’ loyalty and adherence to dietary plans. On HE sites, medicinal properties were attributed to ‘clean’ or ‘pure’ foods, whereas pro-ED sites conveyed their importance for weight reduction. Healthy eating sites were largely entrepreneurial, promoting products or themselves. Pro-eating disorder sites typically featured discussions of bodily disgust, the chastisement of others, and self-discipline in the name of ‘Ana’, such that dieting came to be framed as part of a devotional, often punitive, body project. On both sites, morality discourses were gendered around the thin female body and the ‘ideal mother’, with occasional praise for muscular male bodies. Our findings indicate how transitioning from healthy eating preoccupations to eating disorders may be facilitated by normalising discussions about restrictive dieting and the shaming of bodies, overseen by self-appointed diet ‘experts’ and ‘buddies’ online.

## Introduction

In contemporary society, the internet has become the main platform on which consumers of all ages and backgrounds can seek out information on diet and nutrition, as well as access ‘tips and tricks’ on healthy or restrictive eating (Pollard et al., 2015). Lay and professional people can gain followers and promote products on these websites, while members of forums can talk about what they think to be interesting. What have been called ‘healthy eating’ (HE) websites focus on healthy lifestyle advice and dietary inspiration (Mete et al., 2019), while so-called ‘pro-ED’ (eating disorder) websites and forums allow for discussion of eating ‘problems’ and may encourage or glorify extreme forms of restricted eating (Mento et al., 2021; Overbeke, 2008). Both types of sites serve as communication pathways between ‘experts’ and readers by transforming so-called ‘scientific’ and medical terminology into lay language (Fixsen & Ridge, 2017; Lynch, 2010). However, theories around narrative power suggest that inequalities exist between experts and lay persons, as well as business owners and consumers, in terms of subjective realities created on websites (Foucault & Sheridan, 1970; Plummer, 2020). No known studies have critically examined the power plays, or compared the style of discourses used, on these two website genres, including the linguistic devices and phrases used to instruct members around eating behaviours, body shapes, and lifestyle choices. If, as Foucault states, we need to consider ‘how things work at the level of on-going subjugation’ and the ‘processes which subject our bodies and govern our gestures, dictate our behaviours’ (Foucault, 1976, p. 79), then an in-depth study of discourses taking place on these sites seems overdue. We sought to remedy this situation, by applying critical discourse analysis (CDA) to a broad range of HE and pro-ED sites freely accessible to those navigating the internet. The research questions we sought to answer were: How do the linguistic devices and messages on these two website genres (HE and pro-ED) (1) compare and contrast and (2) shape knowledge, beliefs, and behaviours of users, and encourage transition to more pathological eating behaviours?

### Healthy Eating Websites

Healthy eating websites are interactive and advisory web pages where individuals such as registered dietitians, trainers, and people with an interest in – and/or knowledge of – healthy eating contribute relevant content, typically on a weekly basis. While some healthy eating sites are run through organisations to deliver health education (Lalchandani et al., 2022), many are set up by private individuals for instructive, entertainment, and commercial purposes. Studies suggest that consumers proactively seek out HE sites and that women are their most frequent readers (Ek, 2015; Renahy et al., 2010). In one qualitative study, female readers perceived HE sites written by food professionals to be credible sources of nutrition knowledge, their interactive nature being their most valuable feature (Bissonnette-Maheux et al., 2015). Studies report some health advantages to those visiting HE sites in terms of health literacy (Bensley et al., 2011; Neuenschwander et al., 2013) and improved dietary choice. For instance, a 2014 meta-analysis demonstrated a significant decrease in fat consumption when these kinds of social media platforms were used (Williams et al., 2014).

As modes of disseminating information about healthier lifestyle choices, HE sites appear to align with public health aims (Mete et al., 2019). However, concerns have been raised that some sites potentially normalise disordered behaviours, act as sources of misinformation and scaremongering (Boepple & Thompson, 2014), and make exaggerated claims, in order to attract customers and sell products (Cesiri, 2016). The rise in health consciousness and public suspicions around such things as mass food production as well as artificial flavourings and preservatives (Nielsen, 2019; Ringquist et al., 2016) mean that consumers are often anxious to avoid making ‘wrong’ food choices which could affect their own and their families’ health and well-being (Nicolosi, 2006). When people are concerned about eating the ‘best’ foods, claims made on such sites may be taken as fact. A recent analysis of healthy living, infant-related blogs found that parents were encouraged to create their own feeding formulas, the most frequent ingredient suggested being raw cow’s and goat’s milk, which are associated with foodborne illnesses such as toxoplasmosis and salmonellosis (Davis et al., 2020).

Many HE sites advocate for moderate and flexible approaches to food and diet (Lalchandani et al., 2022). However, studies suggest that some sites encourage orthorexia-type behaviours (Boepple & Thompson, 2014) and amplify idiosyncratic food and body anxieties, adding to the modern disgust of body fat (Donghi & Wennerholm, 2014). The term ‘orthorexia’ refers to a non-DSM/ICD^1^-recognised ED, whereby an individual becomes overly obsessed with the quality and purity of food (Cheshire et al., 2020; Zickgraf, 2020). One major concern of clinicians and dietitians is the rising number of (mostly young) people taking dietary practices to extremes or adopting ‘orthorexic’ practices as a means of covering up an existing eating disorder (ED) such as anorexia nervosa (Fixsen et al., 2020; Musolino et al., 2015).

### ‘Pro-ED’ Websites

Pro-eating disorder forums have been expanding in scope and influence since the early 1990s, although their covert nature makes it difficult to determine their exact number (Mento et al., 2021). Studies exploring the themes and content of pro-ED sites indicate that typically they contain message and discussion boards, blogs, dieting ‘tips and tricks’, body mass index (BMI) calculators, and photo/video galleries providing ‘thinspiration’ for extreme weight loss (Boepple & Thompson, 2016; Stapleton et al., 2019). The overall impact of these sites on vulnerable individuals continues to be debated. Pro-ED sites have garnered criticism by introducing new weight loss strategies (Wilson et al., 2006), often to very young audiences (Christodoulou, 2012; Fairplay, 2022). However, they have also gained some support from feminists as places for identity construction and stigma resistance (Ramos et al., 2011; Yeshua-Katz, 2015).

One issue with these sites concerns their insular nature. In a linguistic exploration of pro-ED sites, users appeared reluctant to form real-life connections (Lyons et al., 2006) – although given the secretive nature of EDs, some individuals may already have been retreating from their usual social networks. In addition to the absence of references to real-life relationships, Wolf and colleagues noted on pro-ED blogs exclamation marks were frequently used to intensify messages and indicate strong self-affirmation, as opposed to question marks that might indicate personal uncertainty or anxieties (Wolf et al., 2013). There remains a possibility that membership to these sites could discourage treatment seeking (Christodoulou, 2012). While one content analysis of pro-ED sites found sites that contained some pro-recovery content, including links to recovery-relevant websites (Borzekowski et al., 2010), most studies, including that by Rouleau and von Ranson (2011), emphasise their ‘anti-recovery’ focus, superficial engagement in pro-treatment options, and promotion of concealment strategies that act as barriers to recovery. Use of religious metaphors on pro-Ana sites has also been highlighted (Day & Keys, 2008; Norris et al., 2006; Stapleton et al., 2019), leading some to compare these contemporary behaviours with those of ‘holy anorexia’ and fasting women, undertaken for purification purposes in the name of religion in times gone by (Dell’Osso et al., 2016). In contrast, self-starvation in contemporary society is widely considered to have been motivated by a culture which sees thinness as ideal and weight as ‘unbearable’ (Bordo, 1993).

### Body Modification and Narrative Power

One way of conceptualising HE and pro-ED platforms is as sites of narrative power (Plummer, 2020). According to Plummer, narrative power is a form of ‘storytelling’ grounded in everyday social activity involving human interaction that competes for meaning-making. For Plummer (2019), narrative power is timeless and ubiquitous. Depending on the context and the relationship between storyteller and receiver, narrative power can be ‘oppressive’ (i.e. employing language to control and coerce behaviours) or ‘empowering’ (i.e. using language as a form of liberation) (Gottschall, 2013; Plummer, 2020, p. 50). Plummer’s concept of ‘narrative power’ chimes with the earlier theories of Foucault (2019a, 2019b) and Fairclough (1992) pertaining to power and discourse. For the purposes of aligning our work with an epistemological position, it is important to distinguish between Foucault’s and Fairclough’s approaches to discourse analysis, especially in the context of power relations. Like Foucault, Fairclough (1992) is interested in the governmental power of discourse over subjects. However, while Foucault studies the archaeological analysis of power, Fairclough focuses more specifically on ‘narrative power’ as constructed through personal narratives and storytelling. Narratives and storytelling are interconnected with the concept of intertextuality which suggests that words and language cannot be viewed as isolated systems (Bakhtin, 1981; Kristeva, 1980); they are structured through social exchange and are embedded with intentions and contexts. Fairclough (1992) emphasised the importance of recognising and understanding intertextuality as a multifaceted phenomenon that plays a crucial role in shaping the way language is used and meanings are constructed in different discourses.

Over and above such points, these sites must be viewed in light of information and communication technologies generating data and creating new marketing opportunities in late capitalist economies. Borrowing from Foucault’s (1977, 2019) ideas about the disciplining of bodies through surveillance, Zuboff (2019) contends that capitalists have the capability to monitor users’ online behaviour, gather data about their preferences, and influence their actions. This concept, termed ‘surveillance capitalism’, encapsulates the idea of businesses shaping user behaviour through extensive data collection and analysis. However, this expansion of the market’s capabilities potentially places children and adolescents at risk online (Potvin Kent et al., 2019). The child-consumer protection organisation ‘Fairplay’ (2022) has called out global platforms such as Instagram for encouraging and profiting from the formation of ED ‘bubbles’ through using algorithms to deliver any content which the platform believes will maximise a user’s time spent on them.

The fact that most users on the sites are women (and young) also raises questions about how messages concerning the disciplining and shaping of girls’ and women’s bodies in contemporary society are promoted. Writing before the advent of social media, Bartky (1988) noted how, under neoliberalism, women’s bodies had been further objectified. This objectivation of the female body has specific consequences for how women look and how they evaluate their bodies, which Bartky associated with the apparent increase in EDs at that time. Studies of pro-ED sites suggest that, in keeping with their largely female audiences, discourses of femininity around body discipline, dietary restriction, and ideal womanhood feature prominently on these sites (Day & Keys, 2008). While this emphasis on thinness has been challenged by factions within the body positivity movement, including third- and fourth-wave (‘cyber’) feminists (Darwin & Miller, 2021), the contemporary desire for skinniness has never really fallen out of fashion (Wiseman, 2022). Looked at from this perspective, women and others using these sites may be participating in discourses around womanhood and body disciplining that ensure that women’s bodies – and potentially those of all genders – are enslaved to the tyranny of dieting and thinness. A critical discourse approach can be used to unmask practices of dominance that take place on websites and to understand how texts in daily life perpetuate social problems by reinforcing chosen ideologies and power relationships (Fairclough, 1992). It is with this in mind that we turn to our description of the methodology used to investigate these different sites and the elements of power within them.

## Methods

Following the methodology of Fairclough, a critical analysis approach was used to examine the messages and the language used on HE and pro-ED websites to disseminate information and shape beliefs and behaviours around eating. Fairclough’s critical realist perspective operates within a framework that seeks to uncover underlying power structures, societal norms, and ideological influences embedded in language (Chouliaraki & Fairclough, 2002). Fairclough’s emphasis on structured analysis and uncovering power relations through linguistic elements often leads to a meticulous examination of specific language choices and their implications for power dynamics and ideological influences within specific contexts. On the other hand, Foucault’s post-structuralist perspective encourages a more abstract exploration of how discourses shape and are shaped by power, aiming to uncover broader patterns and historical contexts in which knowledge and truths are constructed and contested (Arribas-Ayllon & Walkerdine, 2008). Fairclough’s approach tends towards granular scrutiny of language nuances within specific contexts, while Foucault’s approach favours broader analyses, exploring the construction and evolution of discourses within larger societal frameworks. In comparison to Foucault, Fairclough’s approach places greater emphasis on the examination of linguistic devices, aligning with this study’s specific focus on the micro-level. Fairclough’s approach was chosen for its structured approach, facilitating a comprehensive analysis of multiple data sources. Unlike Foucauldian discourse analysis, which can be seen as abstract and better suited for macro-level and political contexts, Fairclough’s method offers a systematic approach tailored to examining the meaning of linguistic choices and nuances present in datasets about everyday life.

### Sample

**Figure 1.**
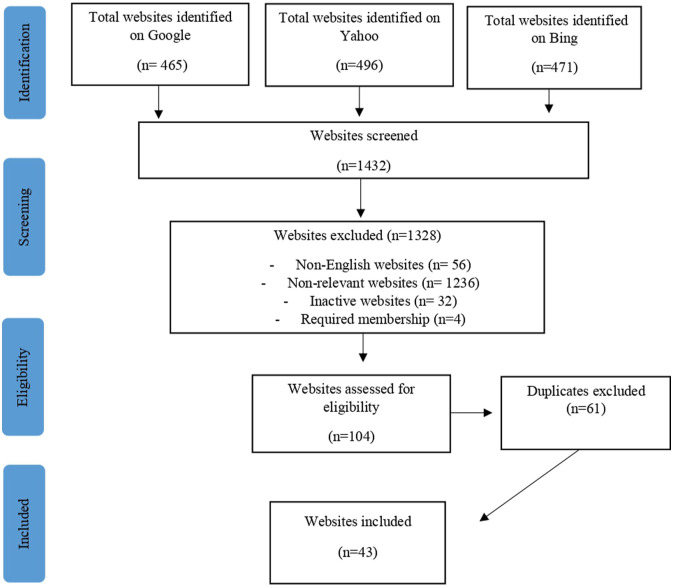
HE website identification strategy.

A purposive sampling approach was followed to identify websites. Personal blogs were excluded from our study to maintain a clear research focus, ensure consistency in data collection, and prevent issues arising of confidentiality breaches and subsequent vulnerability of bloggers. This decision was made considering that highly personal content is often distributed on such blogs. The exact number of websites available on healthy/clean eating sites is difficult to determine (Mento et al., 2021); thus, data selection was concentrated on the first six pages of the most popular three search engines (Google, Yahoo, and Bing). This allowed for some confidence that data saturation could be achieved, since consumer search engine click-through behaviour has shown that users generally do not go beyond the first page of results (Jansen & Spink, 2009). In selecting keywords for our search to identify HE and pro-ED websites, we adopted an approach that aimed to capture a comprehensive range of relevant websites. These keywords were based on established terms commonly used in academic literature, online discussions, and online communities related to the topics. Our keyword selection process involved an analysis of the existing research to identify commonly used terms and phrases associated with HE and pro-ED websites. Additionally, we considered the keywords used in previous studies to ensure comprehensiveness and relevance to our investigation. Data were collected from June 2021 to September 2021. The keywords ‘healthy’, ‘healthy eating’, ‘clean eating’, ‘ortho’, ‘diet’, ‘fitspiration’, ‘fitness’, and ‘fitspo’ were used to identify HE websites. Each site was systematically searched and assessed for eligibility. Websites were deemed eligible if (1) they were written in English, (2) they had a minimum of one post per month, and (3) the content focused on communicating messages about eating to the general population. Websites were excluded if they (1) were only providing healthy recipes, (2) were personal blogs, (3) could not be accessed due to a broken web link, and (4) required a membership account. All results (*n* = 1432), obtained by using the aforementioned keywords on each of the six search engine pages, were collected, and each website was then screened against the inclusion criteria. After screening, the results were tabulated and any duplicates within and across the three search engines were removed, leaving 43 HE websites (Figure 1). Posts that were related to healthy eating were collected for analysis.

**Figure 2.**
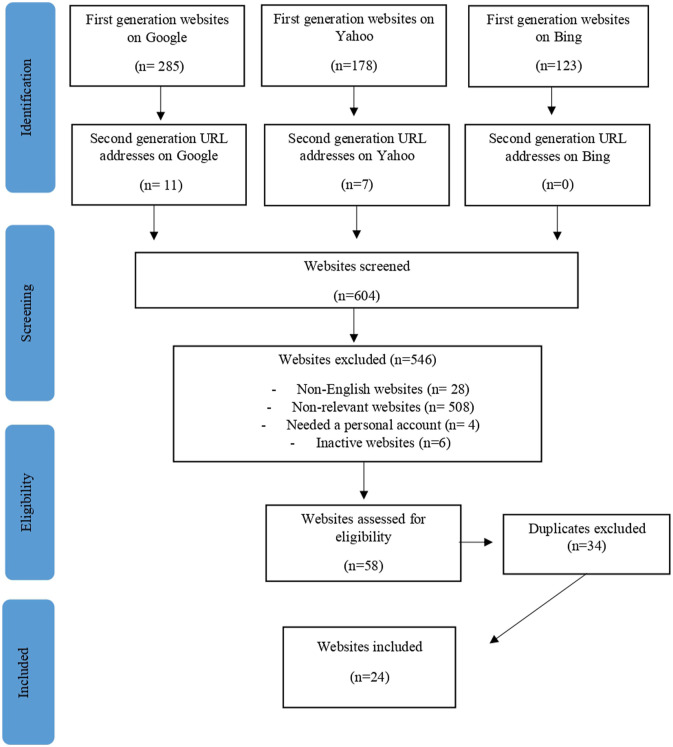
Pro-ED websites identification strategy.

In the same manner, and following the same eligibility criteria for HE website selection, pro-ED websites were identified using the Google, Yahoo, and Bing search engines. In total, 12 key terms (‘proana’, ‘proAna’, ‘proanorexia’, ‘pro-Anorexia’, ‘promia’, ‘proMia’, ‘probulimia’, ‘pro-Bulimia’, ‘proED’, ‘pro-ED’, ‘pro eating disorder’, and ‘pro-eating disorder’) were used to generate a list of pro-ED websites. For the pro-ED sites, the methodology of Borzekowski et al. (2010) was then adopted, and the initial list of sites (‘first’ generation) was used to discover recommended links for additional websites (‘second’ generation). After adding the first- and second-generation websites, 24 pro-ED websites were identified (Figure 2). Threads that contained multiple responses (between 10 and 87) were chosen so that a range of responses and posters could be included in the study.

### Procedure

Ethical permission for the study was gained from the University of Westminster’s Research and Ethics Committee (approval number: ETH2021-2605). Once the websites had been identified, data were collected through careful observation without any interaction with the web moderators or the users, retaining the major advantage of online research, namely its unobtrusiveness. All the data considered for this study constitute open messages that were widely accessible in the public sphere. With the intention to mitigate the risk of disclosing the source or an individual’s identity, identifying information in the data was removed. In a similar vein, URLs or ‘links’ to forum websites are not provided. Finally, extracts were shortened, summarised without losing meaning, or paraphrased to reduce their detectability.

### Data Analysis

The first researcher read the texts from both kinds of sites together and discerned repetitive language such as verbs, adjectives, or phrases that were repeated in several posts/threads. Codes were manually applied to the words/phrases and indicated textual components that were similar across the data. Codes were assessed by the other authors in terms of the degree of similarity and the frequency of their occurrence. Thereafter, they were grouped based on their similarity, ultimately leading to the development of a thematic framework. The data we gathered from the websites presented a rich and multifaceted landscape of information, encompassing various discourses related to diet. Given the complexity of the data, we found that a preliminary thematic framework allowed us to identify the dominant discourses and ideologies and served as a foundation, informing subsequent steps in the CDA process. Fairclough’s CDA, involving description, interpretation, and explanation of discourse, power, and ideology relationships, was then applied in a nuanced and contextually informed manner. The final thematic framework is composed of five main themes: ‘*Expertise in diet and restriction*’; ‘*Health and food fears*’; ‘*Criticism of bodies and motherhood*’; ‘*Reward and punishment*’; and ‘*Discipline and devotion*’.

Once the framework had been identified, Fairclough’s model of CDA was employed. Central to Fairclough’s model (1992) is the aim of raising lay individuals’ consciousness of linguistically exploitative practices in social relations. Fairclough argues that discourse is employed by dominant forces to mould and impose behavioural standards, but the critical element of CDA can unveil hidden truths within the text, informing those who might be in a disadvantaged position (Fairclough, 1992). This method was used to specifically explore ways in which syntactic and grammatical choices are made to support beliefs which potentially trigger particular behaviours, so that language comparisons among websites could be attained. A three-level analysis on each theme was conducted. At the micro-level, the text on each post that communicated the message was examined. At this level, also called textual analysis, the main focus lies on the lexical choices, the aim being to understand why these specific words and phrases were chosen and the attitudes/beliefs that this vocabulary might hinder/engender. For instance, specific adjectives such as ‘beautiful’ and ‘lean’ or metaphors such as ‘couch potato’ and ‘stubborn cushion’ were used to describe the body. The main aim at this level was to understand why these specific words and phrases were chosen and the attitudes that this vocabulary hindered. Thereafter, the purpose at the meso-level was to identify and interpret all the grammatical features and understand how the message/attitude was composed and distributed to readers. The rich lexico-grammatical features were thoroughly recorded, encompassing verb tenses, construction types (active or passive), metaphorical content, use of imperatives, and conditional sentences. These grammatical features provided insights into the authors’ attitudes and intentions. Finally, the social dimensions deriving from both the micro- and meso-levels were interpreted. Consideration of the social dimensions at the macro level was a gradual process as in the textual and grammatical analysis, standards and norms deriving from the lexico-grammatical features were debated and commented on by all authors. It was important to understand the social effects of the messages by making associations between discursive practices and social processes (i.e. understand the changes in human behaviour that might be observed at a larger scale). For instance, an array of messages conveying that some food groups are responsible for health issues and weight gain could enhance a movement of health consciousness and restrictive eating practices.

### Authors’ Statement

In conducting the coding and analysis for this study, we recognise the critical importance of acknowledging our own positionality and its potential influence on the research process. The research team comprised four individuals with shared interests in eating practices, and our variations enriched the analysis by allowing discussion from diverse perspectives. The first author (PT) is a woman, a qualitative researcher with a keen focus on eating behaviours, which evolved from her background as a psychotherapist and her interactions with individuals experiencing an ED. The second (AF) and fourth (AC) authors, also women, have previously conducted research and contributed publications specifically addressing orthorexia, further enhancing our collective expertise in this domain. The third author (DR) is a man, an experienced health-related qualitative researcher, who is relatively new to EDs, and who offered an outsider perspective.

## Results

Through addressing the research question ‘How do the linguistic devices and messages on HE and pro-ED sites compare and contrast in terms of the hooks used in attempts to inform and shape knowledge, beliefs, and behaviours of users?’, the analysis led to five themes, which are discussed in turn below.

### Expertise in Diet and Restriction

While not all authors on HE websites advertised their professional credentials and qualifications in nutrition, establishing the difference between the expert and ‘others’ emerged as an essential element of promoting respect and trust among visitors. This was reinforced through the use of thread titles on these sites that drew attention to people’s expertise (‘*Experts are telling ….*’; ‘*10 things nutritionists want you to know ….*’; and ‘*Here’s what a registered nutritionist says about* ….’). On pro-ED websites, the ‘coaches’/‘experts’ were not introduced as qualified health professionals but people who assumed the role of experts by professing knowledge of techniques for attaining and maintaining low BMI. They claimed expertise based on their success as experienced dieters who had successfully followed a restrictive diet for an extended period. On both forums, to cement their expertise, verbs were used by the ‘experts’ in an instructional manner referring to general actions related to cooking processes or diet such as ‘*cook*’, ‘*add*’, ‘*remove*’, and ‘*opt*’. Other imperative forms were intended to discourage consumption of certain beverages or foods (‘*do not drink calories*’).

Topics such as calorie restriction and keeping a food journal featured on both HE and pro-ED websites, with tables used to indicate the limited quantity of food that one should consume. Calorie restriction and excessive exercise were demanded of users to ‘*get rid of these calories*’ and ‘*calorie budget*’, while commands relating to body weight checks were frequent (‘*check your weight frequently*’). This attitude was semantically legitimised by expert testimonials (‘*we have proof*’) and the instruction to readers to adopt compensatory behaviours (‘*distract yourself*’ and ‘*avoid mirrors*’). Signals of hunger were diminished in value (‘*just because you are hungry*’), while exaggerated generalisations of food restriction with words such as ‘never’ strengthened the relationship between beauty and starvation (‘*No pretty girl eats*’ and ‘*You can never be too thin*’).

### Health and Food Fears

Authors of HE posts were not just disseminating information about healthy eating, they were also trying to instil in reader’s mind the importance of a healthy lifestyle and in many cases sell products. Adverts and affiliated links for Amazon books with low-carb recipes, home bikes, supplements, and weight loss pills were included, claiming to ‘*optimise the user’s performance*’. Commercial content was not identified on pro-ED forums, but a common emphasis on promoting healthy eating was observed across both kinds of sites. The promotion of healthy eating was partly achieved through a kind of ‘medicalisation’ of food – highlighting the link between food quality and illness or health – and through emphasising the social and emotional benefits of having a more desirable or slim body. Authors on HE websites combined verbs in the imperative form with a potentially fatal disease such as cancer to encourage fears about eating ‘wrong’ foods (‘*Eat healthy to avoid cancer*’) and used fighting talk in relation to prevention of serious chronic conditions through a clean diet (‘*Let’s beat type 2 diabetes*’). This linguistic strategy aimed to establish a direct connection between food choices and health outcomes, potentially heightening readers’ anxieties about deviating from website-prescribed dietary norms (‘*If your health is important to you, healthy eating is your answer*’). Claims about lessening the risk of developing a disease through healthy eating were emphasised with exclamations (‘*pssst! That’s your incentive!*’ and ‘*too crucial to overlook!*’) and questions (‘*that seems like a motivation, doesn’t it?*’). This approach to language and emphasis on health outcomes through food choices align with the medicalisation of food, where food becomes not just nourishment but a determinant of health outcomes and well-being.

Authors on pro-ED websites likewise made an association between fasting or restrictive diets and dramatic improvements to physical and dermatological conditions (‘*all your health issues will disappear*’ and ‘*fasting can clear your skin*’). Extreme forms of healthy eating or ‘orthorexic’-related content also frequently featured on pro-ED websites. However, here the aim was less about achieving optimum health and more about avoiding the weight gain pitfalls of ‘unhealthy’, calorific foods. Threads on pro-ED websites were mostly focused on sharing lists of ‘impure’ or ‘safe’ foods (‘*found a really clean brand for safe food*’), with other users frequently asked about their food fears (‘*is anyone else afraid of bananas? Am I crazy?*’). Capital letters (*FEAR*) and exaggerated adjectives such as ‘*major fear*’ were used to indicate the products that one must avoid at one’s peril. The use of the adjective ‘*paranoid*’ suggested an extreme fear and worry of ‘food impurity’ that could potentially encourage users to practice self-starvation (‘*I am paranoid when it comes to plastic packaging*’). Sharing concerns and worries about specific food categories could reinforce fears of food (‘*It is validating to know that I am not the only one with this fear*’).

### Criticism of Bodies and Motherhood

Over and above their health benefits, adopting a restrictive diet was seen as a way of disciplining lazy and flabby bodies and creating a more desirable body shape. On HE websites, the ‘body’ and its parts were widely referenced in posts, through both positive and negative representations. Celebrity endorsement was the key feature identified in relation to male users, conveying muscularity to be the most preferred bodily trait (‘*these six-pack abs and huge muscles*’ and ‘*What Chris Hemsworth does to stay fit?*’). Authors of posts commented on parts of the female body, using positive attributive adjectives for bodies related to healthy eating (‘*defined thighs*’, ‘*beautiful glutes*’, and ‘*lean bodies*’) and negative attributive adjectives for body parts related to ‘unhealthy’ eating (‘*big and bothersome blob*’ and ‘*pesky stomach fat*’). Words with moral connotations, such as ‘*couch potato*’, were employed to further depict negative bodies and similes compared body shape with unpleasant images (‘*Do you want to feel stuffed like a turkey?*’).

To promote relatability, HE websites also included testimonials from ‘ordinary’ people or ‘before and after diet’ pictures of mothers. The linking of ‘good mothering’ with clean or healthy eating was a key feature that was only found on some HE websites and was established through using an informal tone (‘*your kiddo*’). This link was used in association with references to meal prepping as being challenging but emotionally satisfying when done according to the rules of healthy eating. Conditional sentences were also used to emotionally challenge mothers (‘*if you want to feed your kids well*,’ ‘*if you wish to be recognised as a good mother, consider healthy eating*’), the insinuation being that healthy eating would secure the title of the best mom (‘*the number 1 thing to stay on top*’).

No references to the role of mother were found on the pro-ED sites we examined. This is potentially due to the younger age of the pro-ED community. However, it is also plausible that the community avoids explicit mention of motherhood or pregnancy-related terms because these topics are closely tied to the physical changes associated with gestation. The fear of weight gain, which is a central theme within the pro-ED community, could explain the avoidance of motherhood topics. In other respects, pro-ED website users employed similar and sometimes more strongly self-derogating and undermining descriptions for personal body parts, such as ‘*muffin top*’, as well as self-insulting metaphors (‘*I have to get rid of this ugly sack*’) and superlatives (‘*the worst case of muffin top*’) for negative emphasis. In contrast, more positive descriptions of thin bodies were found on pro-ED websites (‘*desirable thigh gap*’ and ‘*flawless thin body*’), suggesting the strong promotion of a thin ideal within these communities. In common, HE and pro-ED authors encouraged their readers to adopt clean eating and restrictive practices for aesthetic reasons, with words like ‘*attractiveness*’, ‘*beauty*’, and ‘*gorgeousness*’ commonly associated with clean and restrictive practices (‘*fasting can clear your skin*’). Zero conditional sentences on pro-ED websites (‘*if you aren’t thin, you are not attractive*’) and comparative adjectives on HE websites (‘*to have a smaller waist*’ and ‘*to have a lower BMI*’) illustrated deeply engrained beliefs that attractiveness is a feature attributed solely to thin individuals. Finally, both types of websites frequently used forms of body shaming, in the form of personifications (‘*cellulite is not cute*’) and evocative verbs (‘*It can ruin your bikini season*’).

### Reward and Punishment

One essential difference between HE and pro-ED sites concerned the ways in which those able to discipline the body via diet were differently rewarded in terms of how the self could be viewed. On HE sites, the adoption of a healthy/clean way of eating was seen as leading to a happier, healthier body. Adjectives such as ‘*healthy*’, ‘*happy*’, and ‘*strong*’ were used by authors to describe the new self, while comparatives (‘*happier*’, ‘*healthier*’, and ‘*stronger*’) constructed a better future self. These comparatives imply that readers will feel better about themselves and will observe positive changes in their self-concept if healthy/clean eating is adopted (‘*you will feel better with yourself*’).

In contrast, on pro-ED sites both ‘experts’ and users often employed the language of punishment, largely in the form of self-hating and sadistic talk. Authors on pro-ED websites’ threads frequently criticised themselves and used derogatory statements in relation to the self. Adjectives such as ‘*fat*’, ‘*worthless*’, ‘*stupid*’, and ‘*useless*’ were used to direct disgust at oneself, but also at others who admitted to feeling hungry. Here, the term ‘*have to*’ expresses the obligation to hate oneself so that hunger can be soothed (‘*you have to hate yourself*’). If the need for food was not contained, self-punishment was considered non-negotiable (‘*if you eat calorific foods, you have to punish yourself*’). In turn, some ‘coaches’ on pro-ED sites boasted of being extremely callous in the methods they used to assist pro-ED members in achieving their weight goals (‘*I am very harsh, mean, strict and controlling*’), while users noted that, for example, ‘*I am ok with punishments*.’ ‘Promises’, such as ‘*I will make your ED worse*,’ ‘*I will hurt you*,’ or ‘*I will traumatise you*’, were made by coaches to attract users who were willing to pay for the cost of skinniness with their well-being.

### Discipline and Devotion

Both HE and pro-ED website posts used different devices to promote a sense of community and loyalty among their visitors and members. The pronoun ‘we’ was often used to convey a sense of understanding (‘*We all know this feeling*’ and ‘*We’ve been there*’) and a sense of support (‘*We’ll keep each other committed*’). In attempts to establish a connection between the authors and their audiences, creators of the sites wrote in the first person and positioned themselves as ordinary individuals, who had also faced challenges with weight (‘*I personally …*’ and ‘*a diet that I even follow*’). Another device used on some HE websites was to create nicknames for the readers who followed the site’s eating challenges or diet tips. In other cases, collective nouns were used to create a distinct identity (‘*Fighters!*’ and ‘*Friends*’). There was a sense that people on pro-ED sites in particular were seeking support from similarly minded individuals, who would keep them focused on their goal (‘*I would like to have some like-minded friends*’ and ‘*we’ll be skinny together*’). Such posts and requests for a ‘*buddy*’ were typically written in an informal tone (‘*Looking for a buddy, preferably around my stats*’).

While those with loyalty to the community and its ethos were welcomed by the HE community, people with opposing views (including friends and family) were often framed as threatening personal integrity and obstructing the dieters’ intentions and goals. Commonly, pro-ED users adopted a dichotomous stance which positioned their community in contrast to otherness. The pronoun ‘we’ was used to establish their distinct identity (‘*we built a community*’ and ‘*many of us*’), which needed to be protected from ‘others’ (‘*be careful of the others*’). References to the others were made when users were advised to conceal their weight loss strategies and avoid treatment.

We also noted a mystic element to the language and messages on these sites. Pledges to be ‘faithful’ were an important requisite of both types of websites. On HE sites, managers asked their readers to make vows and prevail in their combat with ‘demons’ (as in processed and unhealthy food), along with avowals to stick to a chosen dietary path (‘*make a vow to not eat them again*’ and ‘*be faithful to our goal*’). On pro-ED websites, this was more explicitly evidenced using spells, psalms, and rituals to ‘Ana’, who was given the attributes of an entity and praised as a deity-like figure offering protection and guidance (‘*protect me*’ and ‘*guide me through beauty*’). In other ‘psalms’, the ‘god’ of Ana was attributed with punitive qualities and as serving as an agent of behavioural control (‘*try harder*’; ‘*I have a lot of expectations from you*’; and ‘*without my guidance, you will never be thin*’).

## Discussion

**Figure 3.**
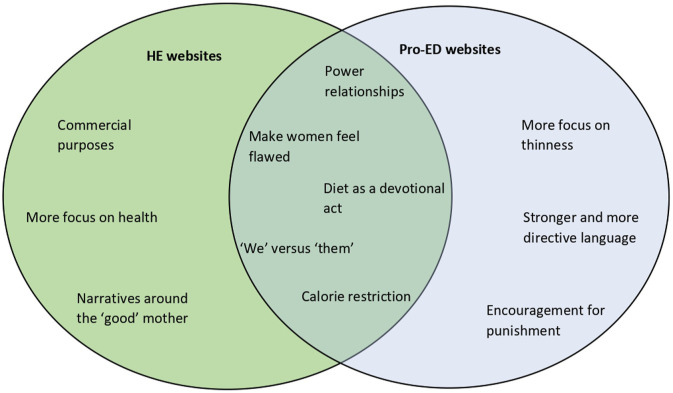
Similarities and differences between HE and pro-ED websites.

We set out to explore and compare the varying discursive styles and language used by authors and message posters on HE and pro-ED websites, to better understand how these sites attempt to shape beliefs and behaviours around eating. Using CDA to critically examine the choice of language and the use of linguistic devices, we were able to gain insights into the types of messages commonly disseminated on these sites and begin to unmask the practices relating to power that take place on such websites. Our analysis is the first to draw attention to the micro-level power relations operating on these two categories of websites, examining their similarities and disparities. Previous studies have looked at the supportive nature and the use of ‘*us*’ versus ‘*them*’ language on sites to promote online in-group identity (Yeshua-Katz, 2015), which, in the present study, was found to be a common feature of both kinds of sites. Despite their differences in orientation – with HE sites more focused on health and commercial sales and pro-ED sites on attaining thinness – HE and pro-ED content shared strong commands to discipline the body through diet and to encourage site visitors to devote considerable time and energy to this project. On HE sites, those with experiences or qualifications in nutrition were positioned as holding narrative power, whereas on pro-ED websites it was those considered to be the most skilled in food restriction and weight loss strategies who dominated discussions. Both types of sites supported traditional ideas about gender, notably, the idea that the slim female body was more desirable and the lean, muscular body was the standard for men. Some HE sites also portrayed an ‘ideal motherhood’ in which both mother and child consume solely healthful, pure food. Self-derogation and negative body talk are widely featured on both types of sites. However, on HE sites, the quest for bodily perfection was framed as part of a healthier lifestyle, whereas on pro-ED sites, there was talk of punishment, bodily disgust, and rigid discipline in the name of ‘Ana’, which at times took on religious connotations (Figure 3).

The remainder of the discussion will focus on the following: *medicalisation and food anxiety*; *devotion and punishment*; *objectifying the female body*; and *power, knowledge, and the internet*.

### Medicalisation and Food Anxiety

There is a popular assumption that the internet has democratised knowledge and culture (Baker, 2022); however, as we have shown, power inequalities operate online. These power inequities are reinforced not only through status effects but also by the use of medical and religious language. Medicalisation has been summarised as the processes by which social phenomena come to be perceived and treated as illnesses or medical matters (Conrad & Schneider, 1980; Halfmann, 2012). On HE sites, hosts instructed visitors and members to take up so-called ‘healthy’ eating as part of lifestyle changes, which largely align with wider circulating ideas around clean eating and health. However, they were also prone to create health fears and make exaggerated claims, with certain foods being presented as a preventative or a cure for serious medical conditions. Medicalisation from above (i.e. via medically trained persons) takes place from a point of clinical training; however, medicalisation from below (i.e. by lay persons (Charland, 2013)) is more likely to be associated with ‘moral panic’ and to be exploited for entrepreneurial purposes (Cohen, 2015). Our previous studies have noted how this kind of medicalisation is linked to orthorexic behaviours including via conversations on the internet (Fixsen & Cheshire, 2022; Fixsen et al., 2020). Studies also suggest that the internet has become a major platform for manufacturing moral panics (Flores-Yeffal & Sparger, 2022). On HE websites, fear is instilled through a constant emphasis on how certain foods are ‘bad’ for both consumers and the planet (Nicolosi, 2006).

Turnwald and colleagues (2022) highlight the frequent emphasis placed on health qualities when healthy food is advertised on online platforms. With discourses on the medicinal properties of food becoming common parlance in commercial settings, the concept of food as medicine can heighten the illusion of safety as well as danger (Poirier, 2016), potentially promoting extreme and pathological healthism (Barthels et al., 2015; Dunn & Bratman, 2016; Fixsen & Cheshire, 2022). On all websites we investigated, conversations about clean and pure foods and their medicinal properties were prominent, with warnings about the pitfalls of dietary deviations. Yet, there was a difference here as, while on HE sites, food and health messages were usually framed in positive terms, on pro-EDs sites clean/pure eating was adopted largely to avoid calorific foods as part of a project of achieving thinness. Nevertheless, our study suggests that at least some HE websites are complicit in perpetuating safety/danger discourses. This is not to say that deliberate deception takes place on these sites but rather that people are frequently searching out cures online, while others are proposing them. The medicalisation of healthy eating and the cultivation of food fears on websites were intricately linked through intertextuality, with discourse surrounding these concepts drawing upon and reinforcing shared narratives and shaping collective beliefs about health and nutrition in a digitally interconnected society. This is happening at a time when data suggests a decline in life expectancies, including in the United States (Heuveline, 2022) and suspicions about environmental pollutants have increased, causing people to look for ways of achieving a longer or more meaningful life (Valente et al., 2020).

### Objectifying the Gendered Body

To understand the general appeal of dietary websites for women in particular, we must consider them in the historical context of the disciplining and beautifying of female bodies in contemporary Western societies. Feminist scholars have long pointed out societal requirements for women to be thin and attractive to be successful, and the dangers this poses in terms of developing an ED (Bartky, 1990; Orbach, 1986). Before the popularity of the internet, Bartky (1990) wrote about the fragmentation and objectification of the female body under neoliberal capitalism, proposing two subjects involved in the process of objectification, the ‘objectifier’ and the ‘objectified person’. On HE and pro-ED sites, the ‘objectifiers’ are likely to be the female authors and the moderators, indicating that women themselves are complicit in promoting stereotypical ideals around female bodies.^2^ More recent studies suggest that young people of all genders, including men and those in the trans community, are increasingly subject to the panic around body shape appearance (Dworkin, 2009; Romito et al., 2021) and therefore likely to be attracted to these sites.

To suggest that this kind of ‘objectification’ of bodies is somehow distinct from identity creation is to dismiss the emotional attachment that people on these sites have to their dietary and body modification projects. For members, this may not represent a fragmentation of self but what they see as a path to self-realisation and fulfilment, or at least one that means far more to them than a cosmetic re-shaping of the body. In this sense, their aim is not just to ‘correct’ their body through restriction and surgical or non-surgical procedures; rather, it is also to achieve a level of gratification by negotiating their self-identity within these sites. This supports other findings, which suggest that people do find meaning and purpose through these sites (Gale et al., 2016; Yeshua-Katz & Martins, 2013), even if these goals appear unwholesome or harmful from a psychological perspective. We suggest that the disciplining of the female body in the context of these sites is one which fits with wider notions of power and identity tied in with neoliberalism and its goals of entrepreneurial activity and self-striving. There are many who think that young people are being put at danger due to the huge reach and influence of pro-ED sites (Fairplay, 2022). In the case of pro-Ana, the quest for thinness often manifests as a kind of slavery to an ultimately self-defeating goal.

### Devotion and Punishment

Belonging has the capacity to satisfy the human need for acceptance, but where the group is advocating activities that are antisocial or harmful, it may threaten the health of individuals (Allen et al., 2021). While a sense of community was cultivated on the websites we reviewed, so too was a kind of cultism linked with diet and foods. Linguistically, this was chiefly achieved through the use of collective nouns and attribution of group identity to those following proscribed diet plans, with the exclusion of others who failed to follow these rules. On HE websites, processed food was frequently demonised, with readers asked to make vows to abstain from it. On pro-ED sites, this was taken further: abstinence from food and weight loss were clearly framed as a form of worship. In this way, the dietary project becomes metaphysical, with sacrifices and punishments required. The use of chastising and praising messages on sites could, we suggest, also reinforce the loyalty of members to these sites.

The ways in which pro-ED sites encourage members to pursue ‘Ana’ as a devotional path have been discussed in other studies (Day & Keys, 2008; Stapleton et al., 2019), and our findings provide further evidence of this. We also noted how on pro-ED sites both ‘experts’ and users frequently employed the language of punishment and self-hate, and sometimes promoted sadistic language to reinforce the group and individual commitment to the shared end goal of weight loss. This appears to be a two-way process: the power of those making the commands on these sites was reinforced by the behaviours of (the usually) young female users searching out coaches. They appeared eager to conform to these harsh and seemingly punitive rules, the implication being that the end results would be worth it. Why this devotional element occurs is unclear, but it appears to link to both historical associations between self-starvation and saintliness (Dell’Osso, 2016) and clinical literature associating EDs with self-harming behaviours (Sansone & Levitt, 2002). Conformity to the rules of the ‘good anorectic’ (Day & Keys, 2008, p. 14; Eckermann, 1997) has always been part of pro-Ana. However, it is possible that the language used on the sites may be becoming more directive and extreme, in efforts to counter the medical and pro-health messages discouraging this kind of extreme eating.

### Power, Knowledge, and the Internet

Whatever the motivation is for people seeking out these sites, our study suggests that they function as places for power plays between so-called experts, experienced users, and novices. From a discourse analysis perspective, we were able to observe how power manifested through certain forms of language. For example, website use of repetitious, exaggerated, or emotive language encouraged visitors to adopt certain beliefs and behaviours which may override prevailing attitudes and advice, such as about eating a varied diet or keeping within recommended BMI weight. Foucault argues that language and knowledge equal power; hence, those with the knowledge exercise power (Foucault, 1991). The instructional effect (Cesiri, 2016) in our study was magnified through the use of imperatives which commanded, advised, and requested others to think, behave, or conform in particular ways (Jary & Kissine, 2014; Pennock-Speck & Fuster-Márquez, 2014).

Studies suggest that individuals’ movement to Orthorexia Nervosa (ON) tendencies can be initiated by time spent on healthy eating blogs and social media sites (Mitrofanova et al., 2021). It seems likely that on both HE and pro-ED sites, discourses around bodies and diet will be largely imparted or overseen by ‘experts’ (often self-appointed), ‘educating’ and instructing website users in ways to discipline their minds and bodies. Drawing on these ideas, the discourses on websites can be seen as forms of intertextuality, including reflections of prior contexts and discourses that have shaped individuals’ understanding of ‘good’ and ‘bad’ food or lean bodies and ‘best’ identities. Also, it is likely that at least some of these ‘expert’ accounts will be written from personal experiences or anecdote, thereby potentially competing with medically sanctioned discourses around health, identity, and ideal body weight and shape.

Foucault recognised that practices of self-surveillance and self-discipline were more powerful when individuals perceived them as actions of their own free will and of self-care (Foucault, 1979, 2001). The self-care projects promoted here were made interesting and compelling through advertising and corporate messages, but also through creating packages of self-care activities, that can be promoted on these sites, and which provide income to local entrepreneurs. Our study confirms how, in the interests of self-care, people are prepared to invest considerable time, attention, and money in various dietary and fitness regimes. Power and knowledge in the form of specific and tailored forms of ‘self-care’ were thence transferred to the recipients of these messages, potentially challenging or even replacing more institutionally sanctioned narratives about bodies, health, and food.

Finally, we must also look at contemporary society’s ever-expanding online technology to understand how our relationship to food, our bodies, and our health has, and is, being fashioned (Baker, 2022). What Zuboff (2015) calls ‘surveillance capitalism’ is now a dominant feature of our social world. By this, Zuboff means the widespread collection and commodification of personal data by corporations, including search histories, social media posts, physical locations, and product keywords. Surveillance capitalism has allowed for the unprecedented commodification of personal information first through corporations collecting information, such as preferences and online interactions without the user’s consent (Lawrence, 2018; Zuboff, 2015), and then subsequently by selling this data to others for commercial uses. Information on users’ online behaviour allows corporations to powerfully tailor advertising in order to shape our preferences. In macro terms, the economics of self-care are shaped by companies like Google and Instagram, with their ability to detect and dictate choices. On the micro-scale, linguistic and visual devices are used in local power plays on these sites to encourage specific self-care practices which fit with the branding of these sites.

### Study Strengths and Limitations

Our study focused on women, and while this allowed a deep examination of the issues for this group, analysis of discourses related to LGBTQ communities and men were restricted. Although a small quantity of posts/threads related to men were identified, websites were mainly addressed to women and messages were focused primarily on the female body and womanhood, and it is likely that some men as well as LGBTQ members use these sites and are exposed to these messages. Despite the stereotypical notion that pro-ED sites are mainly female communities, engagement by men in these sites has been found, highlighting the internal contradictions around masculinity and social norms (Quiniones & Oster, 2019). The perpetuated stigma around men and EDs, as well as the limited provision of support services, has increased male participation on these sites (Quiniones & Oster, 2019). Future studies informed by our findings could uncover further insights into the messages specifically tailored to men and LGBTQ communities. Healthy eating and pro-ED sites can be currently found in many languages, thus allowing for further analyses and interpretations. Given that our study included only those written in English, claims regarding the full spectrum of sites cannot be made. That said, English is a relatively global language, and users from different countries were interacting on these sites.

A strength of our study lies in our attempt to identify and analyse a large quantity of sites, both HE and pro-ED, our comparing and contrasting of the linguistic content of the sites, and our highlighting of messages and the discursive styles that might trigger or amplify concerns over food and bodies. We have also added to the feminist debate on the disciplining of bodies versus self-realisation, the ambiguities on these messages, and potential exploitation of young women through mass media.

### Conclusions

On dietary forums, knowledge topics around personal experiences and know-how relating to health and eating attempt to confer power over others who choose to visit them. As we have emphasised, the so-called ‘experts’ on these sites are also subject to circulating discourses, including around diet, body ideals, and entrepreneurism. It requires the cooperation and willingness of participants to embrace these messages and beliefs, and there are bigger agents complicit in this process, including the social media giants and advertising companies gathering and using data for personal profiling. Diet on these forums takes the form of a devotional act that requires discipline and self-mortification, reflecting a spiritual process in which punishments are permitted. This paper is likely to be valuable to scholars specialising in linguistics, health psychology, the sociology of medicine, and others interested in the topic of healthy and restrictive eating. We aimed to contribute to the ongoing analysis of these websites, deciphering the multifaceted messages disseminated by authors, moderators, and users on these sites. Mental health professionals may also benefit from our research, which might enhance understanding of the messages circulating on these websites and the potential for treatment implications. By considering these phenomena, the ways in which individuals on online platforms potentially transition from moderate to extreme eating behaviours can be better understood by policy makers attempting to break the cycle of transition from healthy to more pathological forms of eating behaviours. In such ways, discourse analysis reaches beyond academia to inform wider policy.

## Notes

1. DSM is the abbreviation used for the Diagnostic and Statistical Manual of Mental Disorders. ICD is the abbreviation used for the International Classification of Diseases (ICSD).

2. The ideal male body on these sites was generally depicted as being more muscular on the sides, suggesting a stereotypical gendering of body types. However, due to little content being found on this topic, male experiences have not formed part of the discussion.
